# Comparative effectiveness of ciltacabtagene autoleucel in CARTITUDE‐1 versus physician's choice of therapy in the Flatiron Health multiple myeloma cohort registry for the treatment of patients with relapsed or refractory multiple myeloma

**DOI:** 10.1002/jha2.312

**Published:** 2021-12-10

**Authors:** Thomas Martin, Amrita Krishnan, Kwee Yong, Katja Weisel, Maneesha Mehra, Sandhya Nair, Keqin Qi, Anil Londhe, Joris Diels, Concetta Crivera, Carolyn C. Jackson, Yunsi Olyslager, Martin Vogel, Jordan M. Schecter, Arnob Banerjee, Satish Valluri, Saad Z. Usmani, Jesus G. Berdeja, Sundar Jagannath

**Affiliations:** ^1^ UCSF Helen Diller Family Comprehensive Cancer Center San Francisco California USA; ^2^ Judy and Bernard Briskin Center for Multiple Myeloma Research Duarte California USA; ^3^ University College Hospital London UK; ^4^ University Medical Center Hamburg‐Eppendorf Hamburg Germany; ^5^ Janssen Global Services LLC Raritan New Jersey USA; ^6^ Janssen Pharmaceutica NV Beerse Belgium; ^7^ Janssen R&D LLC Titusville New Jersey USA; ^8^ Janssen Scientific Affairs LLC Horsham Pennsylvania USA; ^9^ Levine Cancer Institute‐Atrium Health Charlotte North Carolina USA; ^10^ Janssen R&D Raritan New Jersey USA; ^11^ Sarah Cannon Research Institute Nashville Tennessee USA; ^12^ Mount Sinai Medical Center New York New York USA

**Keywords:** CARTITUDE‐1, ciltacabtagene autoleucel, Flatiron Health, indirect treatment comparison, relapsed or refractory multiple myeloma, triple‐class exposed

## Abstract

**Introduction:**

Ciltacabtagene autoleucel (cilta‐cel) is a novel chimeric antigen receptor T‐cell therapy that is being evaluated in the CARTITUDE‐1 trial (NCT03548207) in patients with relapsed or refractory multiple myeloma (RRMM) who received as part of their previous therapy an immunomodulatory drug, proteasome inhibitor, and an anti‐CD38 monoclonal antibody (i.e., triple‐class exposed). Given the absence of a control arm in CARTITUDE‐1, this study assessed the comparative effectiveness of cilta‐cel and physician's choice of treatment (PCT) using an external real‐world control arm from the Flatiron Health multiple myeloma cohort registry.

**Methods:**

Given the availability of individual patient data for cilta‐cel from CARTITUDE‐1 and PCT in Flatiron, inverse probability of treatment weighting was used to adjust for unbalanced baseline covariates of prognostic significance: refractory status, cytogenetic profile, International Staging System stage, time to progression on last regimen, number of prior lines of therapy, years since diagnosis, and age. Comparative effectiveness was estimated for progression‐free survival (PFS), time to next treatment (TTNT), and overall survival (OS). A range of sensitivity analyses were conducted.

**Results:**

Baseline characteristics were similar between the two cohorts after propensity score weighting. Patients with cilta‐cel had improved PFS (HR: 0.18 [95% CI: 0.12, 0.27; *p *< 0.0001]), TTNT (HR: 0.15 [95% CI: 0.09, 0.22; *p *< 0.0001]), and OS (HR: 0.25 [95% CI: 0.13, 0.46; *p *< 0.0001]) versus PCT. Cilta‐cel treatment benefit was robust and consistent across all sensitivity analyses.

**Conclusion:**

Cilta‐cel demonstrated significantly superior effectiveness over PCT for all outcomes, highlighting its potential as an effective therapy in patients with triple‐class exposed RRMM.

## 1 INTRODUCTION

Multiple myeloma (MM) is an incurable disease with a high rate of relapse [1]. Treatment often involves sequential lines of therapy (LOTs) with three commonly used classes of agents: immunomodulatory agents (IMiDs), proteasome inhibitors (PIs), and monoclonal antibodies (MoABs) [2, 3]. Most patients ultimately become refractory to the major classes of MM therapy, leaving few treatment options and a limited survival. Currently, there is no standard of care for patients with relapsed or refractory multiple myeloma (RRMM) who are triple‐class exposed (to IMiDs, PIs, and anti‐CD38 MoABs), with previous evidence showing that this population is treated with at least 336 different regimens that comprise 40 different compounds[4], frequently consisting of continuous triplet therapies [5]. Despite this heavy treatment burden, outcomes for these patients remain poor, with median progression‐free survival (PFS) ranging from three to six months and median overall survival (OS) less than 12 months [2, 6].

Ciltacabtagene autoleucel (cilta‐cel; JNJ‐68284528) is a novel chimeric antigen receptor T‐cell (CAR‐T) therapy with two B‐cell maturation antigen (BCMA)‐targeting single‐domain antibodies currently under evaluation for the treatment of patients with triple‐class exposed RRMM in the CARTITUDE‐1 clinical trial (NCT03548207) [7]. Results indicate substantial activity and duration of response, which appear to be more favorable than currently available treatments [7, 8]. However, given the heterogeneity in currently used treatments and the observed poor outcomes in triple class exposed patients, no randomized clinical trials have compared cilta‐cel with any of these treatments directly.

Due to the absence of a control arm in CARTITUDE‐1, there is a need for indirect evidence on the relative effectiveness of cilta‐cel versus treatments used in current clinical practice. Indirect treatment comparisons (ITCs) are possible using real‐world (RW) data from patients in the MM cohort registry of the Flatiron Health (FH) longitudinal database. ITC methods can align these patients with the CARTITUDE‐1 population, creating an external, RW control arm. This enables a hypothetical head‐to‐head trial comprising both datasets, in which the observed CARTITUDE‐1 cohort would represent patients randomized to cilta‐cel, and the RW cohort would represent patients who would have been randomized to physician's choice of treatment.

In this study, ITCs were conducted to retrospectively evaluate the comparative effectiveness of cilta‐cel from CARTITUDE‐1 and physician's choice of treatment from a RW dataset in patients with triple‐class exposed RRMM.

## 2 METHODS

### 2.1 Data sources

#### 2.1.1 CARTITUDE‐1

CARTITUDE‐1 is an open‐label, single‐arm, Phase Ib/II trial evaluating the safety and efficacy of cilta‐cel in adult patients with triple‐class exposed RRMM. Patients in the United States (US) cohort of CARTITUDE‐1 were recruited between July 2018 and October 2019 at multiple centres. Upon enrolment, all patients underwent leukapheresis to provide T‐cells to generate the cilta‐cel treatment. A comprehensive overview of the CARTITUDE‐1 study has been previously published [7]. The present analysis was based on an updated data cut‐off of February 2021, representing a median follow‐up of 18 months [8].

#### 2.1.2 Flatiron Health database

FH's longitudinal database consists of deidentified patient‐level electronic health records from US community‐based oncology clinics and academic centers. The database includes a MM cohort registry of approximately 10,000 patients from over 280 clinics, representing mainly community‐based oncology practices [9]. Patients in this cohort were newly diagnosed with MM (ICD‐9 203.0x or ICD‐10 C90.0x, C90) and had at least two documented clinical visits on or after January 1, 2011 [9]. The FH MM cohort registry uses abstraction technology to collect both structured and unstructured data, which provide detailed information on demographic characteristics, clinical characteristics such as International Staging System (ISS) stage, treatment histories, and progression data. Patients included in the present analysis initiated eligible LOTs between February 2016 and December 2019 and were followed until February 2021 to match the follow‐up in CARTITUDE‐1, corresponding to a median follow‐up period of 21.9 months.

### 2.2 Study population and design

This study used individual patient‐level data from the CARTITUDE‐1 clinical trial and deidentified, RW data from the FH MM cohort registry. The treated population of CARTITUDE‐1, which consisted of all patients who were infused with cilta‐cel (occurring a median of 47 days after apheresis), was used in the main analysis. To create an external control arm for CARTITUDE‐1 (referred to as the RW cohort), patients from the FH MM cohort registry were included in the present analysis if they satisfied key eligibility criteria for CARTITUDE‐1 (i.e., triple‐class exposed, at least three prior LOTs,^1^ Eastern Cooperative Oncology Group [ECOG] score less than two, creatinine less than or equal to 2 mg/dL,^2^ and disease progression within 12 months of the most recent LOT). Patients in the RW cohort must also have received at least one subsequent treatment after triple class exposure. The RW cohort was adjusted to align with the baseline characteristics of CARTITUDE‐1 using average treatment effect in the treated (ATT) weights derived from propensity scores. This emulated a hypothetical head‐to‐head trial comprising both datasets, wherein the observed CARTITUDE‐1 cohort represented patients randomized to cilta‐cel, and the RW cohort represented patients randomized to physician's choice of treatment.

Given that the FH MM cohort registry is a retrospective database, it was possible to include patients in the current analysis at the earliest LOT initiated after all key eligibility criteria of CARTITUDE‐1 were met. In contrast, patients in CARTITUDE‐1 may have received additional LOTs between the time at which they first met all eligibility criteria and the time at which they were enrolled into CARTITUDE‐1. To account for this difference, patients in the RW cohort who received multiple subsequent therapies after meeting eligibility criteria contributed multiple observations (corresponding to all eligible LOTs) to the current analysis, provided they met eligibility criteria at the beginning of each LOT. The use of all eligible LOTs is supported by previous literature, which found that this approach provides the most statistical efficiency compared to including only the first or last eligible LOT [10, 11]. An exploratory analysis using only the first eligible LOT for patients in the RW cohort was conducted to assess potential differences in results from the two approaches.

To ensure comparability between study cohorts, it was necessary to account for the median of 47 days between apheresis and cilta‐cel infusion in CARTITUDE‐1, thereby avoiding survivorship bias in favor of cilta‐cel [10]. To do so, treatment lines were excluded from the RW cohort if disease progression or death occurred within 47 days of initiating the respective LOT. The index dates were defined as the date of cilta‐cel infusion for treated patients in CARTITUDE‐1 and as 47 days after initiating the relevant treatment for each observation in the RW cohort.

### 2.3 Baseline characteristics for population alignment

Differences between nonrandomized cohorts in baseline characteristics that are prognostic of outcomes may bias comparative effectiveness estimates if left unadjusted [12]. In this study, prognostic factors for adjustment were chosen using a clinician‐driven process. First, a list of potential factors was identified a priori by consulting studies from a literature review conducted to identify clinical outcomes in triple‐class exposed RRMM patients. This list was presented to a panel of clinical experts and modified according to their input. The panel was then asked to rank each variable in order of importance for adjustment. To aid in this process, clinicians were provided with univariate regression results showing the prognostic strength of each variable in terms of PFS and OS in CARTITUDE‐1. Clinicians were also provided with the standardized mean difference (SMD) for each factor between CARTITUDE‐1 and the RW cohort (an SMD ≤ 0.1 was considered a small difference, an SMD > 0.1 and ≤ 0.2 a moderate difference, and an SMD > 0.2 a substantial difference [13]). Clinician rankings were revised iteratively until consensus was achieved. The panel determined that refractory status, cytogenetic profile, ISS stage, time to progression on last regimen, number of prior LOTs, years since MM diagnosis, and age were the minimal set of covariates that should be adjusted for to ensure clinical validity of the analyses. Hence, these variables were adjusted for in the base case analysis. Total plasmacytoma was also among the most important factors but was not available from the FH database and therefore was not included. The remaining identified variables where rank‐ordered from most to least important (Table S1).

### 2.4 Outcomes

Outcomes of interest were PFS, time to next treatment (TTNT), and OS. In CARTITUDE‐1, PFS was calculated as the time from the index date to disease progression or death, whichever occurred first. For patients who had not progressed and were alive at data cut‐off, data was censored at the last disease evaluation before the start of any subsequent antimyeloma therapy or the retreatment of cilta‐cel. Conversely, as progression data may be less strictly monitored and hence more likely to be missing in RW data than in clinical trials, PFS was defined in the RW cohort as the time from the index date to the date of progression, death, or start of next treatment, whichever occurred first, with the date of last follow‐up used in censoring. TTNT was defined as the time from the index date to the initiation of the next LOT or death, whichever occurred first. Patients who were still alive and had not initiated a new LOT at the data cut‐off were censored at the last date known to be alive. OS was defined as the time from the index date to the date of the patient's death. If the patient was still alive or their vital status was unknown, data were censored at the last date known to be alive (CARTITUDE‐1) or the last follow‐up date (maximum of last treatment end date or last visit date) (RW cohort). The middle of the month was used as the date of death in the RW cohort because only month and year of death were available in the FH database. Mortality data in the FH database is derived by amalgamating multiple data sources and has been validated against the National Death Index [14].

### 2.5 Statistical methods

Inverse probability of treatment weighting (IPTW) was used to balance baseline characteristics between patient populations [15]. First, propensity scores were calculated using a logistic regression model that predicted assignment in the CARTITUDE‐1 cohort as a function of baseline covariates. ATT weighting was applied, with patients in the CARTITUDE‐1 cohort kept as observed (i.e., assigned a weight of one) and patients in the RW cohort receiving a weight of *p*/(1–*p*), where *p* is the propensity score predicting inclusion in the CARTITUDE‐1 cohort [15]. Patients in the RW cohort with similar characteristics to that of the observed CARTITUDE‐1 population received larger weights, thereby balancing the two cohorts. The effective sample size (ESS) was calculated to reflect the impact of weighting on the available information in the individual patient‐level data [16].

Estimates of comparative effectiveness were derived for both the unadjusted comparison (i.e., cilta‐cel versus physician's choice of treatment prior to IPTW), and the adjusted comparison (i.e., with IPTW). A Cox proportional hazards model (with weights applied for the adjusted comparison) was used to estimate the hazard ratio (HR) and its respective 95% confidence interval (CI) or PFS, TTNT, and OS. The selected covariates were additionally adjusted for in the model for doubly robust results [17]. The cluster‐robust sandwich variance–covariance estimator was used to account for within‐person clustering of observations, as all eligible treatment lines for the RW cohort were included in the main analysis. The validity of the proportional hazards assumption was assessed based on visual inspection of the log‐cumulative hazard plot, visual inspection of the Schoenfeld residuals plot, and the Grambsch–Therneau test [18], with a *p*‐value less than 0.05 considered to indicate a violation of the assumption.

For the RW cohort, variables with missing values (ISS stage, hemoglobin, lactate dehydrogenase, and ECOG score) were imputed using the closest reported value prior to the index date. If no value was reported, multiple imputation with chained equations was used. Imputation for the CARTITUDE‐1 data was not required since it did not have missing values. All statistical analyses and graphical interpretation of the results were carried out with SAS 9.4 (SAS Institute, Cary, North Carolina) and R version 4.0.3 (R Foundation for Statistical Computing, Vienna, Austria).

#### 2.5.1 Sensitivity analyses

Five distinct sensitivity analyses were conducted to assess the effect of varying the patient populations, statistical methods, handling of missing data, variables, and inclusion criteria. In each sensitivity analysis, only one analytic specification was modified, with all other specifications aligning with those previously outlined for the main analysis in the Methods. A comprehensive summary of the analytic specifications for each sensitivity analysis is reported in Table S2.

The first sensitivity analysis included all enrolled patients who underwent apheresis in CARTITUDE‐1, including those who discontinued prior to receiving treatment. This analysis also waived the requirement of no disease progression or death within 47 days of initiating the relevant LOT in the RW cohort. The second analysis used multivariable regression that adjusted for base case covariates in the model in place of IPTW. The third analysis excluded the observations from the RW cohort with incomplete information for the base case variables (only ISS contained missing values). The fourth analysis adjusted for the following variables: hemoglobin level, lactate dehydrogenase level, prior stem cell transplant, ECOG status, race, sex, and type of MM, in addition to the base case variables. The fifth analysis applied additional inclusion criteria to the RW cohort to align with the adequate organ function that is a general requirement for patients enrolled in clinical trials. In this analysis, the RW cohort was restricted to patients who had hemoglobin values of at least 8 g/dL and platelet counts of at least 50 × 10^9^ per liter.

## 3 RESULTS

### 3.1 Adjustment for imbalances between cohorts

**FIGURE 1:**
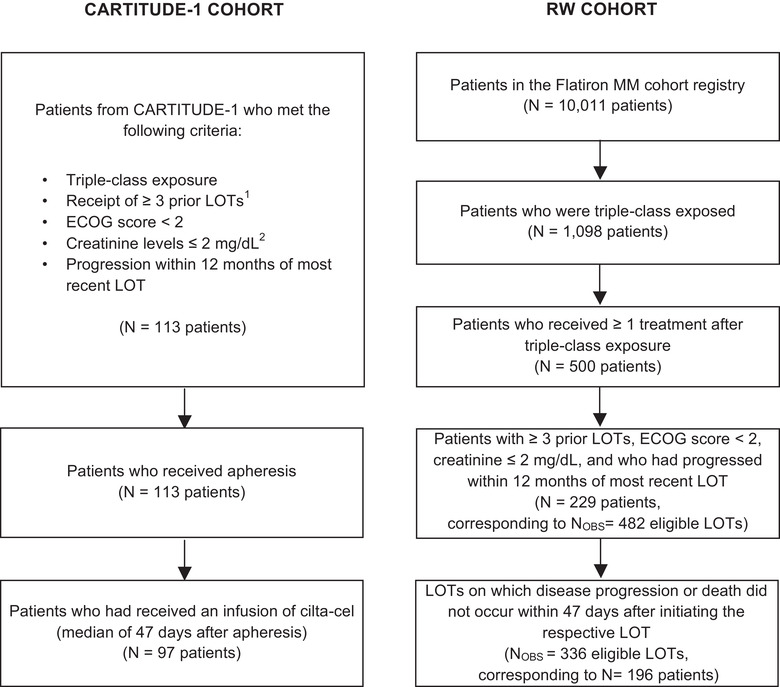
Flow chart of patient selection [1]. CARTITUDE‐1 inclusion criteria required at least three prior LOTs or double refractoriness to an immunomodulatory drug and a proteasome inhibitor; however, all enrolled patients received at least three prior LOTs [2]. CARTITUDE‐1 inclusion criterion was creatinine clearance of ≥40 mL/min/1.73 m^2^; however, all enrolled patients had creatine levels ≤ 2 mg/dL. Abbreviations: ECOG, Eastern Cooperative Oncology Group; LOT, line of therapy; MM, multiple myeloma; *N*_OBS,_ number of observations; RW, real world

The main analysis comprised two patient cohorts: the treated population in CARTITUDE‐1 (*N* = 97) and 196 patients from the FH MM cohort registry (i.e., the RW cohort). Patients in the RW cohort contributed a total of 336 observations across all eligible LOTs (Figure 1). Baseline characteristics before and after adjustment with IPTW for the base case variables are given in Table 1. Prior to adjustment, substantial differences (SMD > 0.2) between the cohorts were observed for all available base case variables, with the CARTITUDE‐1 population having a greater proportion of patients with ISS stage I, high‐risk cytogenetics (at least one of del17p, t[4;14], or t[14;16]), progression within 4 months on their most recent LOT, four or fewer prior LOTs, and patients younger than 65 years of age. In contrast, compared to CARTITUDE‐1, the RW cohort had a greater proportion of patients who were penta‐refractory (to at least two IMiDs, at least two PIs, and an anti‐CD38 MoAB) or who had been diagnosed with MM less than 6 years prior. After adjustment, alignment between the two populations was improved, with the mean SMD reduced from 0.32 to 0.10. A summary of SMDs for each variable before and after adjustment is shown in Figure S1.

**TABLE 1:** Overview of baseline characteristics before and after adjustment with IPTW for the base case

		Unadjusted		Adjusted
Variable	Categories	CARTITUDE‐1, *N* (%) 97 (100%)	RW cohort, *N*_OBS_ (%) 336 (100%)	RW cohort, ESS^a^ (%) 80 (100%)
*Variables used in adjustment with IPTW for the base case*				
Refractory status^b^	Penta refractory^c^ Triple or quad refractory^d^ Others	41 (42.3) 44 (45.4) 12 (12.4)	177 (52.7) 130 (38.7) 29 (8.6)	37 (46.3) 36 (45.5) 7 (8.2)
ISS stage	I II III	61 (62.9) 22 (22.7) 14 (14.4)	120 (35.7)^e^ 106 (31.5)^e^ 110 (32.7)^e^	53 (66.7) 16 (20.6) 10 (12.7)
Cytogenetic profile	High risk^f^ Standard risk Unknown	23 (23.7) 68 (70.1) 6 (6.2)	62 (18.5) 181 (53.9) 93 (27.7)	17 (21.8) 58 (72.1) 5 (6.1)
Time to progression on last regimen	≤4 months >4 months	48 (49.5) 49 (50.5)	101 (30.1) 235 (69.9)	40 (50.5) 40 (49.5)
Presence of total plasmacytoma^g^	No Yes	78 (80.0) 19 (20.0)	NR NR	NR NR
Number of prior LOTs	≤4 >4	33 (34.0) 64 (66.0)	65 (19.3) 271 (80.7)	24 (29.8) 56 (70.2)
Years since MM diagnosis	<6 ≥6	45 (46.4) 52 (53.6)	259 (77.1) 77 (22.9)	38 (47.6) 42 (52.4)
Age	<65 ≥65	62 (63.9) 35 (36.1)	129 (38.4) 207 (61.6)	51 (63.2) 29 (36.8)
*Variables not used in adjustment with IPTW for the base case*				
Hemoglobin (g/dL)	<12 ≥12	90 (92.8) 7 (7.2)	249 (74.1)^h^ 87 (25.9)^h^	59 (74.1) 21 (25.9)
LDH levels (units/L)	<280 ≥280	85 (87.6) 12 (12.4)	284 (84.5)^i^ 52 (15.5)^i^	67 (83.4) 13 (16.6)
Prior stem cell transplant	No Yes	10 (10.3) 87 (89.7)	137 (40.8) 199 (59.2)	21 (24.5) 60 (75.5)
ECOG status	0 1	39 (40.2) 58 (59.8)	98 (29.2)^j^ 238 (70.8)^j^	33 (40.8) 50 (58.5)
Race	White Black/African American Not reported/other	69 (71.1) 17 (17.5) 11 (11.3)	233 (69.3) 48 (14.3) 55 (16.4)	57 (71.5) 13 (16.3) 10 (12.2)
Sex	Female Male	40 (41.2) 57 (58.8)	151 (44.9) 185 (55.1)	32 (39.6) 48 (60.4)
Type of MM	IgG Light chain Other	57 (58.8) 24 (24.7) 16 (16.5)	198 (58.9) 58 (17.3) 80 (23.8)	48 (59.5) 16 (20.3) 16 (20.2)

^a^ ESS was rounded to whole numbers.

^b^ Refractoriness was defined as discontinuation of drug of interest within 60 days and starting a different drug in the next line or starting a new drug within 60 days after end of previous treatment (RW cohort) and by International Myeloma Working Group consensus criteria (CARTITUDE‐1) [7, 21].

^c^ Refractory to at least two IMiDs, two PIs, and an anti‐CD38 MoAB.

^d^ Refractory to two IMiDs and one PI; or two PIs and one IMiD; or two IMiDs and two PIs.

^e^ ISS stage was imputed for 96 observations in the RW cohort.

^f^ At least one of del17p, t(14;16), or t(4;14).

^g^ Includes extramedullary plasmacytomas and soft‐tissue components of bone‐based plasmacytomas [24].

^h^ Hemoglobin was imputed for 1 observation in the RW cohort.

^i^ LDH was imputed for 97 observations in the RW cohort.

^j^ ECOG was imputed for 84 observations in the RW cohort.

**Abbreviations**: ATT, average treatment effect in the treated; ECOG, Eastern Cooperative Oncology Group; ESS, effective sample size; IMiD, immunomodulatory drug; IPTW, inverse probability of treatment weighting; ISS, International Staging System; LDH, lactate dehydrogenase; LOTs, lines of therapy; MM, multiple myeloma; MoAB, monoclonal antibody; *N*_OBS_, number of observations; NR, not reported; PI, proteasome inhibitor; RW, real world; SMD, standardized mean difference.

Physician's choice of treatment received in all eligible LOTs consisted of 51 different regimens. Treatments received either alone or as part of combination therapies included IMiDs (pomalidomide, lenalidomide, and thalidomide), PIs (carfilzomib, ixazomib, and bortezomib), and MoABs (daratumumab and elotuzumab). See Table 2 for more details on the treatments that comprised physician's choice.

**TABLE 2:** Physician's choice of treatment received across all eligible lines of therapy in the RW cohort

Treatments	Hierarchy^a^ *N* (%) 336 (100%)	Received in any eligible line of therapy^b^ *N* (%) 336 (100%)
Carfilzomib	98 (29)	98 (29)
Pomalidomide	69 (21)	97 (29)
Daratumumab	28 (8)	54 (16)
Ixazomib	20 (6)	29 (9)
Elotuzumab	21 (6)	43 (13)
Bortezomib	30 (9)	61 (18)
Lenalidomide	7 (2)	55 (16)
Panobinostat	1 (0)	4 (1)
Selinexor	11 (3)	17 (5)
Thalidomide, or melphalan, or cyclophosphamide^c^	15 (4)	83 (25)
Dexamethasone alone	5 (1)	5 (1)
Others^d^	31 (9)	64 (19)

^a^ For each treatment, the number and percent represent the patients who received that treatment as a single‐agent therapy or in combination with any of the other treatments listed in the subsequent rows.

^b^ Received alone or in combination; therefore, the total adds to more than 100% as treatments from the same line of therapy can be counted more than once.

^c^ Any one received alone or in combination with either one of the three or other drugs.

^d^ “Others” included bendamustine, cisplatin, doxorubicin, etoposide, decitabine, fludarabine, ibrutinib, venetoclax, and clinical study drug.

**Abbreviation**: RW, real world.

### 3.2 Comparative effectiveness results

Comparative effectiveness estimates for cilta‐cel versus physician's choice of treatment in both the unadjusted and adjusted analyses for PFS, TTNT, and OS are shown in Table 3. Prior to adjustment, the HR for cilta‐cel versus physician's choice of treatment was 0.20 (95% CI: 0.14, 0.28; *p *< 0.0001), 0.17 (95% CI: 0.11, 0.24; *p *< 0.0001), and 0.28 (95% CI: 0.18, 0.45; *p* < 0.0001) for PFS, TTNT, and OS, respectively. After adjustment, cilta‐cel significantly reduced the risk of disease progression or death by approximately 82% (HR: 0.18 [95% CI: 0.12, 0.27; *p *< 0.0001]), the risk of receiving a subsequent treatment by approximately 85% (HR: 0.15 [95% CI: 0.09, 0.22; *p *< 0.0001]), and the risk of death by approximately 75% (HR: 0.25 [95% CI: 0.13, 0.46; *p *< 0.0001]).

**TABLE 3:** Estimated medians and comparative effectiveness for cilta‐cel versus physician's choice of treatment

	Median, months (95% CI)			Hazard ratio^a^ (95% CI), *p*‐value for cilta‐cel vs. physician's choice of treatment	
	CARTITUDE‐1	RW cohort			
	Unadjusted	Unadjusted	Adjusted^b^	Unadjusted	Adjusted^b^
PFS	22.8 (22.8, NR)^c^	4.47 (3.78, 5.03)	4.50 (2.40, 5.85)	0.20 (0.14, 0.28), <0.0001	0.18 (0.12, 0.27), <0.0001
TTNT	NR (NR, NR)	4.93 (4.27, 5.52)	4.53 (2.86, 6.77)	0.17 (0.11, 0.24), <0.0001	0.15 (0.09, 0.22), <0.0001
OS	NR (23.6, NR)	14.78 (12.29, 17.84)	13.24 (9.17, 21.29)	0.28 (0.18, 0.45), <0.0001	0.25 (0.13, 0.46), <0.0001

^a^ HR < 1 indicates favorable treatment effect for cilta‐cel.

^b^ Adjusted for refractory status, International Staging System stage, cytogenetic profile, time to progression on last regimen, number of prior lines of therapy, years since multiple myeloma diagnosis, and age.

^c^ Median should be interpreted with caution, as reached when few patients were still at risk and may be an underestimate.

**Abbreviations**: CI, confidence interval; HR, hazard ratio; NR, not reached; OS, overall survival; PFS, progression‐free survival; RW, real world; TTNT, time to next treatment.

**FIGURE 2:**
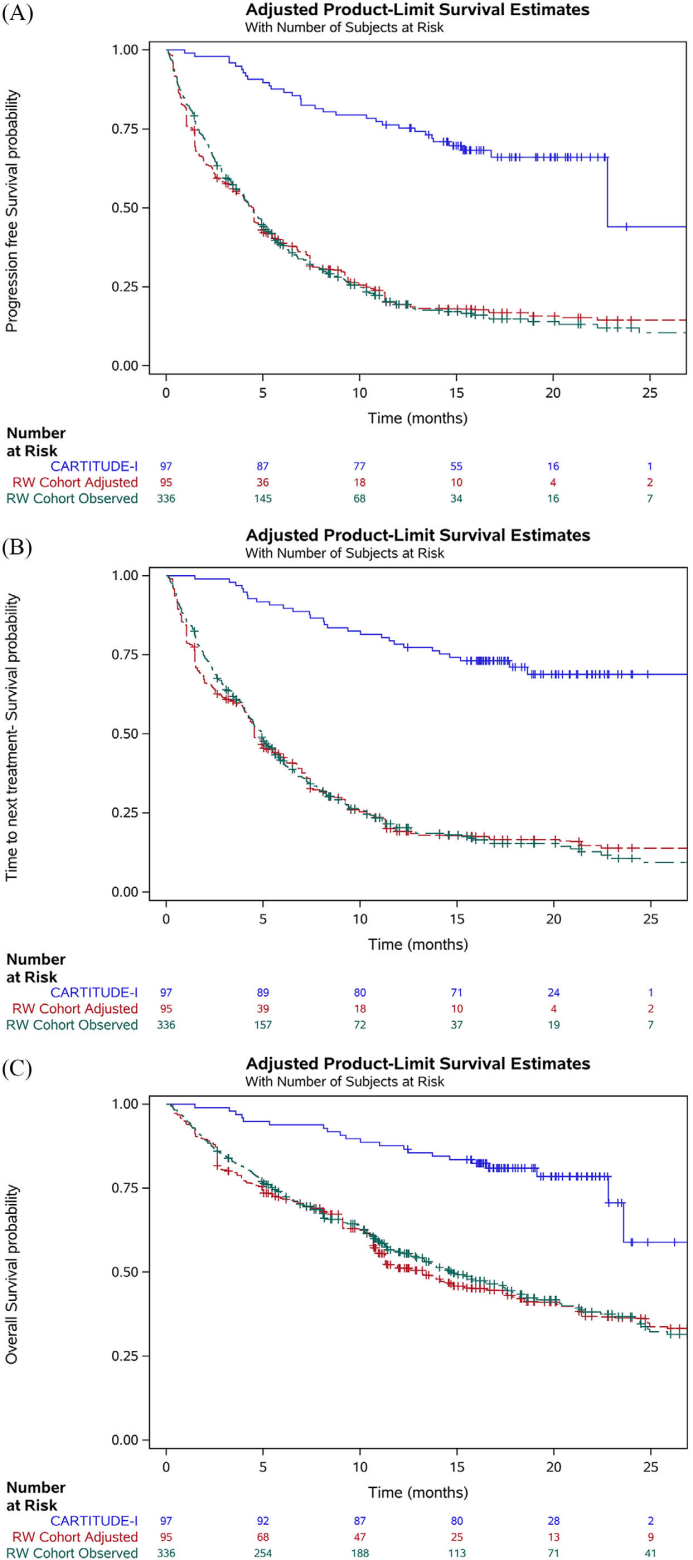
Kaplan–Meier plots for (A) progression‐free survival, (B) time to next treatment, and (C) overall survival in CARTITUDE‐1 (observed) and the RW cohort (observed and adjusted). Note: Number at risk for the adjusted RW cohort represents the sum of the propensity score weights, not the effective sample size. Adjusted results correspond to the base case analysis which adjusted for refractory status, International Staging System stage, cytogenetic profile, time to progression on last regimen, number of prior lines of therapy, years since multiple myeloma diagnosis, and age. The adjusted curves reflect inverse probability of treatment weighting with average treatment effect in the treated weights (not doubly robust). Abbreviation: RW, real world

Duration of PFS, TTNT, and OS for cilta‐cel exceeded physician's choice of treatment (Figure 2). For patients treated with cilta‐cel, median TTNT and median OS were not reached, whereas median PFS was 22.8 months (95% CI: 22.8, not reached) but was observed at a timepoint where few patients remained at risk and is therefore expected to be underestimated. For patients treated with physician's choice of treatment, the median PFS and TTNT were reached within 5 months, and median OS within 15 months, both before and after adjustment (Table 3). The results of the Grambsch–Therneau test [18] for proportional hazards assumption were found to be nonsignificant for each outcome (PFS: *p *= 0.16; TTNT: *p *= 0.08; OS: *p *= 0.27), indicating that the proportional hazards assumption was not violated.

The exploratory analysis that considered only the first eligible LOT for patients in the RW cohort produced similar results to those from the main analysis (Table 4).

**TABLE 4:** Comparative effectiveness of cilta‐cel versus physician's choice of treatment across additional analyses

			Exploratory analysis	Sensitivity analyses				
		Main analysis	First eligible LOT only for patients in the RW cohort	1. Enrolled population of CARTITUDE‐1	2. Multivariable regression	3. Complete case	4. Adjustment for all variables	5. Additional inclusion criteria from CARTITUDE‐1 applied to the RW cohort
*Sample size*								
CARTITUDE‐1	*N*	97	97	113	97	97	97	97
RW cohort	*N*_OBS_ (ESS)	336 (80)	196 (32)	482 (192)	336	240 (49)	336 (50)	262 (60)
*Outcome*								
PFS	HR^a^ (95% CI) *p*‐value	0.18 (0.12, 0.27) <0.0001	0.15 (0.09, 0.24) <0.0001	0.21 (0.14, 0.32) <0.0001	0.18 (0.12, 0.27) <0.0001	0.14 (0.09, 0.22) <0.0001	0.15 (0.09, 0.24) <0.0001	0.16 (0.11, 0.25) <0.0001
TTNT	HR^a^ (95% CI) *p*‐value	0.15 (0.09, 0.22) <0.0001	0.13 (0.08, 0.21) <0.0001	0.18 (0.12, 0.27) <0.0001	0.14 (0.09, 0.22) <0.0001	0.12 (0.07, 0.18) <0.0001	0.12 (0.07, 0.20) <0.0001	0.14 (0.09, 0.21) <0.0001
OS	HR^a^ (95% CI) *p*‐value	0.25 (0.13, 0.46) <0.0001	0.22 (0.11, 0.44) <0.0001	0.32 (0.19, 0.55) <0.0001	0.26 (0.15, 0.46) <0.0001	0.26 (0.13, 0.53) 0.0002	0.17 (0.08, 0.35) <0.0001	0.24 (0.12, 0.47) <0.0001

The main analysis included the following specifications: all eligible LOTs from the RW cohort, treated population of CARTITUDE‐1, inverse probability of treatment weighting, missing values imputed, and adjustment for base case variables (refractory status, cytogenetic profile, International Staging System stage, time to progression on last regimen, number of prior lines of therapy, years since multiple myeloma diagnosis, and age). For each additional analysis, one of these specifications were modified, as outlined in Table S2.

^a^ HR < 1 indicates favorable treatment effect for cilta‐cel.

**Abbreviations**: CI, confidence interval; ESS, effective sample size; HR, hazard ratio; LOT, line of therapy; *N*_OBS_, number of observations; OS, overall survival; PFS, progression‐free survival; RW, real world; TTNT, time to next treatment.

### 3.3 Sensitivity analyses

In the sensitivity analysis that included the enrolled population of CARTITUDE‐1, results were aligned with those from the main analysis, with HRs of 0.21 (95% CI: 0.14, 0.32; *p *< 0.0001), 0.18 (95% CI: 0.12, 0.27; *p *< 0.0001), and 0.32 (95% CI: 0.19, 0.55; *p *< 0.0001), for PFS, TTNT, and OS, respectively (Table 4). The results of all other sensitivity analyses were also consistent with the main analysis, with cilta‐cel demonstrating a significant improvement over physician's choice of treatment for all outcomes.

## 4 DISCUSSION

Cilta‐cel, a novel CAR‐T therapy targeted at BCMA, demonstrated early, deep, and durable responses and a manageable safety profile for patients with triple‐class exposed RRMM in CARTITUDE‐1 [7, 19]. Direct comparisons of cilta‐cel and other treatment regimens have not been made, as the feasibility and ethics of comparative trials are complicated by a myriad of factors, including patients’ advanced disease stage; the relatively small number of potential trial participants; the lack of a clear standard of care for patients with triple‐class exposed RRMM [5]; and the complex nature of CAR‐T therapies, which makes it difficult to administer meaningful control treatments. In the absence of a comparator arm, an external control arm from a RW data source such as FH's longitudinal database can be used to estimate the relative therapeutic effects of cilta‐cel versus physician's choice of treatment. However, such estimates can be biased if there are meaningful differences in baseline characteristics between the patient populations. It is therefore critical to ensure nonrandomized populations being assessed indirectly are well‐balanced across all variables potentially prognostic of outcomes. Hence, ITCs using IPTW methods were used to generate unbiased comparative effectiveness data that can inform decisions by both clinicians and payers.

The present study derived indirect comparisons between cilta‐cel, as assessed in CARTITUDE‐1, and conventional treatments used in RW clinical practice, as assessed in the FH MM cohort registry. Propensity score methods were used to align the RW cohort with the population of CARTITUDE‐1. Results for PFS, OS, and TTNT were all statistically significant in favor of cilta‐cel in the main analysis, clearly demonstrating its superior effectiveness and clinical value. The exploratory analysis that considered only the first eligible LOT for patients in the RW cohort, rather than all eligible LOTs, produced a negligible change in the results. Furthermore, results were robust to an array of sensitivity analyses that aimed to assess the appropriateness of the populations, methods, and variables used. For instance, cilta‐cel continued to demonstrate favorable results after broadening the inclusion criteria to all enrolled CARTITUDE‐1 patients. Results also remained consistent with the main analysis when multivariable regression was used in place of IPTW; when patients with missing values were excluded to assess the impact of imputation; and when additional prognostic factors were considered. Overall, the magnitude and consistency of the present findings across all sensitivity analyses suggest that cilta‐cel represents a promising new treatment option for patients with triple‐class exposed RRMM.

The strength of the control arm used in the present study provided additional confidence in the findings. The RW cohort represented a contemporaneous external control for CARTITUDE‐1, with a long‐term median follow‐up of 21.9 months. The RW cohort comprised US‐based patients, similar to the patients included from the CARTITUDE‐1 study. Additionally, the median OS, PFS, and TTNT observed in the RW cohort were on the upper range of those that have been observed in other RW cohorts of RRMM patients [2, 6, 20, 21]. In the unadjusted RW cohort, the median OS, PFS, and TTNT were 14.78 months (95% CI: 12.29, 17.84), 4.47 months (95% CI: 3.78, 5.03), and 4.93 (95% CI: 4.27, 5.52), respectively. In contrast, a multicenter study conducted by the International Myeloma Working Group in 2017 reported a median OS of 13.0 months (95% CI: 11.0, 15.0) for patients who had received at least three prior LOTs and were double‐refractory to an IMiD and a PI but not necessarily triple class exposed [20]. Patients who received a subsequent therapy after fulfilling eligibility criteria had a median PFS of 5.0 months (95% CI: 4.0, 6.0) [20]. Additionally, the retrospective MAMMOTH study, which had a data cut‐off of 2018, reported a median OS of 9.3 months (95% CI: 8.1, 10.6) and a median PFS of 3.4 months (95% CI: 2.8, 4.0) for patients who received a subsequent treatment after becoming refractory to an index regimen containing an anti‐CD38 MoAB [2]. One study of a US‐based, RW cohort of triple‐class exposed patients reported a median time to discontinuation of 4.2 months (95% CI: 3.1, 5.2) [22].

As in any nonrandomized study, the potential for residual confounding cannot be excluded. However, the availability of individual patient‐level data from both cohorts enabled adjustment for imbalances in important prognostic factors. To ensure that the most important clinical factors were balanced between the two populations, an evidence‐informed process—which incorporated published literature, clinical opinion, prognostic strength of variables, and baseline differences between the study cohorts—was used to select the covariates for adjustment. In this study, differences in factors included in the base case were minimal after weighting, strengthening the validity of the comparison. Furthermore, compared to other oncology databases, FH provided a comprehensive range of baseline characteristics, optimizing data availability for the present analysis [9]. Of all prognostic factors identified a priori, total plasmacytomas (including extramedullary plasmacytomas) was the only variable that was unavailable for the RW cohort. Moreover, it has been reported that the frequency of extramedullary disease in MM patients increases during the course of the disease [23]. Hence, it is most likely that the patients in the RW cohort had less extramedullary disease given that they were not as heavily pre‐treated as the CARTITUDE‐1 patients, which is a potential bias against CARTITUDE‐1.

The use of RW data presents inherent limitations. For instance, the FH database did not provide data on response outcomes such as overall response rate and complete response or better rate, preventing comparative effectiveness analyses of these outcomes. Information on comorbidities was also unavailable from the FH database. Furthermore, monitoring of patients in RW databases is less rigorous and subject to greater variation than in clinical trials, where patients are regularly and strictly monitored. This is especially relevant for PFS outcomes, as progression data were more likely to be missing for patients in the RW cohort than in CARTITUDE‐1. To address this, the earliest of start of a new LOT or disease progression was considered a progression event in the RW cohort, as start of a new LOT may have been more reliably reported than progression. This may have overestimated the time to progression for patients in the RW cohort, as the next treatment is expected to be initiated after progression. Thus, the true benefit conferred by cilta‐cel on PFS may be even greater than that observed in the present analyses. Alternatively, the modified PFS definition may have misclassified progression for patients who initiated a new LOT for reasons other than progression; however, at this late stage in a patient's treatment journey, efficacy (i.e., progressive disease) is most often the reason for initiating a new LOT. OS data quality was less of a concern, as methods to assess OS have been validated for the FH MM cohort registry [14].

Future analyses using real‐world data can confirm findings from the current analyses. Such studies will also help to better understand the safety of cilta‐cel versus physician's choice of treatment, which was outside of the scope of the present study. Furthermore, even though cilta‐cel has superior efficacy to real‐world conventional treatments in this study period, similar to other CAR‐Ts, cilta‐cel can only be delivered in certified specialized treatment centers. Thus, real‐world evidence can also provide insight into the referral patterns related to CAR‐T therapies.

## 5 CONCLUSION

The present study assessed the comparative effectiveness of cilta‐cel versus treatments received by a similar cohort of patients in RW clinical practice. Cilta‐cel demonstrated statistically and clinically superior results for all outcomes studied (PFS, TTNT, and OS), and these were robust across a range of sensitivity analyses. Based on these results, cilta‐cel offers substantial clinical benefits for patients with triple‐class exposed RRMM compared with physician's choice of conventional treatment.

## AUTHOR CONTRIBUTIONS

All authors were responsible for study conception and design. AL, KQ, and SN were responsible for data acquisition and analysis. All authors were responsible for interpretation of data and revising it critically.

## CONFLICT OF INTEREST

KW received honoraria from and served in a consulting or advisory role for Adaptive Biotechonlogies, Amgen, BMS, Celgene, GSK, Janssen, KaryopharmTherapeutics, Oncopeptides, Roche/Genentech, Sanofi, and Takeda, served in a consulting or advisory role for GSK, received travel funding from Amgen, BMS, Celgene, GSK, Janssen, and Takeda, and received research funding from Amgen, Celgene, Janssen, and Sanofi.

TM served in a consulting or advisory role for GlaxoSmithKline and Juno Therapeutics, and received research funding from Amgen, Janssen, and Sanofi.

AK served in a consulting or advisory role for Adaptive Biotechnologies, Celgene/Bristol Meyers‐Squibb, GlaxoSmithKline, Janssen Oncology, Pfizer, Regeneron, served on speakers bureaus for Amgen, Celgene/Bristol Meyers‐Squibb, GlaxoSmithKline and Takeda, served on scientific advisory boards for Sutro Biopharma, has equity in Celgene/Bristol Meyers‐Squibb, and received research funding from Janssen Oncology.

SJ is a consultant for Bristol Myers Squibb, Janssen, Karyopharm Therapeutics, Merck, Sanofi, and Takeda Pharmaceuticals.

SZU served in a consulting or advisory role for AbbVie, Amgen, Celgene, GlaxoSmithKline, Janssen, Karyopharm Therapeutics, Merck, Seattle Genetics, Skyline Diagnostics, and Takeda, served on speakers bureaus for Celgene, Janssen, Sanofi, and Takeda, and received research funding from Amgen, Array BioPharma, Bristol Myers Squibb, Celgene, GlaxoSmithKline, Janssen, Merck, Pharmacyclics, Sanofi, Seattle Genetics, and Skyline Diagnostics.

JGB served in a consulting or advisory role for Bluebird Bio, Bristol Myers Squibb, Celgene, CRISPR Therapeutics, Janssen, Karyopharm Therapeutics, Kite/Gilead, Legend Biotech, Secura Bio, Servier, and Takeda, and received research funding from AbbVie, Acetylon Pharmaceuticals, Amgen, Bluebird Bio, Bristol Myers Squibb, Celgene, Celularity, Constellation Pharmaceuticals, CURIS, EMD Serono, Genentech/Roche, Glenmark, Ichnos Sciences, Janssen, Kesios Therapeutics, Lilly, Novartis, Poseida, Sanofi, Takeda, Teva, and Vivolux.

KY is consultant physician and received honoraria from Janssen, GSK, Amgen Inc., Takeda, Sanofi and research funding from Janssen, Takeda, Sanofi.

AL, CC, JMS, KQ, MV, AB, JD, SN, SV, and YO are employed by Janssen and have restricted stock units and/or stock options. CCJ is employed by Janssen and is a consultant physician at the Memorial Sloan Kettering Cancer Center. MM was employed by Janssen when the study was conducted.

## FUNDING

This work was supported by Janssen Pharmaceuticals and Legend Biotech.

## Supporting information

Appendix

## Footnotes

1 CARTITUDE‐1 inclusion criteria required at least three prior LOTs *or* double refractoriness to an IMiD and a PI; however, all enrolled patients received at least three prior LOTs.

2 CARTITUDE‐1 inclusion criterion was creatinine clearance of ≥40 mL/min/1.73 m^2^; however, all enrolled patients had creatine levels ≤ 2 mg/dL.
