# Supplementary material for: Comparative effectiveness of ciltacabtagene autoleucel in CARTITUDE‐1 versus physician's choice of therapy in the Flatiron Health multiple myeloma cohort registry for the treatment of patients with relapsed or refractory multiple myeloma
Source: EJHaem. 2021 Dec 10;3(1):97–108. doi: 10.1002/jha2.312 (PMC9175662; doi:10.1002/jha2.312)
Supplement: Supplementary file 1 — Appendix [file JHA2-3-97-s001.docx]

# SUPPLEMENTAL APPENDIX

**Title**: Comparative effectiveness of ciltacabtagene autoleucel in CARTITUDE-1 versus physician’s choice of therapy in the Flatiron Health multiple myeloma cohort registry for the treatment of patients with relapsed or refractory multiple myeloma

**Authors**: Thomas Martin^1^*, Amrita Krishnan^2^, Kwee Yong^3^, Katja Weisel^4^, Maneesha Mehra^5^, Sandhya Nair^6^, Keqin Qi^7^, Anil Londhe^7^, Joris Diels^6^, Concetta Crivera^8^, Carolyn C. Jackson^10^, Yunsi Olyslager^6^, Martin Vogel^5^, Jordan M. Schecter^9^, Arnob Banerjee^9^, Satish Vallur^5^, Saad Z. Usmani^10^, Jesus G. Berdeja^11^, Sundar Jagannath^12^

**Affiliations & E-mail Address**: ^1^UCSF Helen Diller Family Comprehensive Cancer Center, San Francisco, CA, USA; ^2^Judy and Bernard Briskin Center for Multiple Myeloma Research, City of Hope, Duarte, CA, USA; ^3^University College Hospital, London, UK; ^4^University Medical Center Hamburg-Eppendorf, Hamburg, Germany; ^5^Janssen Global Services, LLC, Raritan, NJ, USA; ^6^Janssen Pharmaceutica NV, Beerse, Belgium; ^7^Janssen R&D, LLC, Titusville, NJ, USA; ^8^Janssen Scientific Affairs, LLC, Horsham, PA, USA; ^9^Janssen R&D, Raritan, NJ, USA; ^10^Levine Cancer Institute-Atrium Health, Charlotte, NC, USA; ^11^Sarah Cannon Research Institute, Nashville, TN, USA; ^12^Mount Sinai Medical Center, New York, NY, USA

**Corresponding Author**: Thomas Martin

**Contacts**: Thomas Martin, UCSF Helen Diller Family Comprehensive Cancer Center Communications Department, Box 0981, UCSF San Francisco, CA 94143-0981. Email: Tom.Martin@ucsf.edu

**Funding Source**: This research was supported by Janssen Pharmaceuticals and Legend Biotech.

Table S1: Ranking of prognostic factors and availability in CARTITUDE-1 and RW Cohort

| Variable | Rank | Available in CARTITUDE‑1? | Available in RW Cohort? | Categories |
| --- | --- | --- | --- | --- |
| *Variables for base case* | | | | |
| Refractory status^1^ | **Required*** | Yes | Yes | Penta refractory: at least 2 IMiDs, 2 PIs, and an anti-CD38 MoAB  Triple or quad refractory: 2 IMiDs and 1 PI; or 2 PIs and 1 IMiD; or 2 IMiDs and 2 PIs  Others |
| Cytogenetic profile | **Required*** | Yes | Yes | High Risk: at least one of del17p, t(4;14), or t(14;16)  Standard Risk  Unknown |
| ISS stage | **Required*** | Yes | Yes | I  II  III |
| Total plasmacytoma^2^ | **Required*** | Yes | No | NA |
| Time to progression on last regimen | **Required*** | Yes | Yes | ≤ 4 months  > 4 months |
| Number of prior LOTs | **Required*** | Yes | Yes | ≤ 4  > 4 |
| Years since MM diagnosis | **Required*** | Yes | Yes | < 6 ≥ 6 |
| Age (years) | **Required*** | Yes | Yes | < 65  ≥ 65 |
| *Other identified variables in order of importance* | | | | |
| Hemoglobin | 9 | Yes | Yes | < 12 g/dL  ≥ 12 g/dL |
| LDH levels | 10 | Yes | Yes | < 280 units/L  ≥ 280 units/L |
| Prior stem cell transplant | 11 | Yes | Yes | No  Yes |
| ECOG status | 12 | Yes | Yes | 0  1 |
| Race | 13 | Yes | Yes | White  Black/ African American  Not Reported / other |
| Sex | 14 | Yes | Yes | Female  Male |
| Type of MM | 15 | Yes | Yes | IgG  Light Chain  Other |

***** Variables labelled “required” were considered equally important by clinical experts.
^1^ Refractoriness was defined as discontinuation of drug of interest within 60 days and starting a different drug in the next line or starting a new drug within 60 days after end of previous treatment (RW cohort) and by International Myeloma Working Group consensus criteria (CARTITUDE-1).^1,2^
^2^ Includes extramedullary plasmacytomas and soft-tissue components of bone-based plasmacytomas.^3^

**Abbreviations:** ECOG, Eastern Cooperative Oncology Group; IMiD, immunomodulatory drug; ISS, International Staging System; LDH, lactate dehydrogenase; LOTs, lines of therapy; MM, multiple myeloma; MoAB, monoclonal antibody; NA, not applicable; PI, proteasome inhibitor; RW, real-world

**Table S2: Detailed overview of additional analyses**

|  | **Main Analysis** | **Exploratory Analysis** | **Sensitivity Analyses** | | | | |
| --- | --- | --- | --- | --- | --- | --- | --- |
|  |  | **First Eligible LOT Only for Patients in the RW Cohort** | **1. Enrolled Population of CARTITUDE-1** | **2. Multivariable Regression** | **3. Complete Case** | **4. Adjustment for All Variables** | **5. Additional Inclusion Criteria from CARTITUDE-1 Applied to the RW Cohort** |
| **CARTITUDE-1 Population** | All treated patients | All treated patients | All enrolled patients | All treated patients | All treated patients | All treated patients | All treated patients |
| **Inclusion Criteria for RW Cohort** | Key criteria from CARTITUDE-1^1^ + no PD or death ≤ 47 days of index date^2^ | Key criteria from CARTITUDE-1^1^ + no PD or death ≤ 47 days of index date^2^ | Key criteria from CARTITUDE-1^1^ | Key criteria from CARTITUDE-1^1^ + no PD or death ≤ 47 days of index date^2^ | Key criteria from CARTITUDE-1^1^ + no PD or death ≤ 47 days of index date^2^ | Key criteria from CARTITUDE-1^1^ + no PD or death ≤ 47 days of index date^2^ | Key criteria from CARTITUDE-1^1^ + no PD or death ≤ 47 days of index date^2^+ Hgb ≥8 g/dL and platelets ≥50 × 10^9^/L |
| **Statistical Method** | IPTW with ATT weights (doubly robust) | IPTW with ATT weights (doubly robust) | IPTW with ATT weights (doubly robust) | Multivariable regression | IPTW with ATT weights (doubly robust) | IPTW with ATT weights (doubly robust) | IPTW with ATT weights (doubly robust) |
| **Included LOTs for Patients in the RW Cohort** | All eligible LOTs | Patients’ first eligible LOT only | All eligible LOTs | All eligible LOTs | All eligible LOTs | All eligible LOTs | All eligible LOTs |
| **Handling of Missing Data** | Imputation | Imputation | Imputation | Imputation | Observations with missing values for covariates of interest were excluded | Imputation | Imputation |
| **Covariates** | Base case^3^ | Base case^3^ | Base case^3^ | Base case^3^ | Base case^3^ | Base case^3^ + Hgb, LDH, prior SCT, ECOG status, race, sex, and type of MM | Base case^3^ |
| **Outcomes** | PFS, OS, TTNT | PFS, OS, TTNT | PFS, OS, TTNT | PFS, OS, TTNT | PFS, OS, TTNT | PFS, OS, TTNT | PFS, OS, TTNT |

^1^ Key criteria from CARTITUDE-1 were applied as follows: triple-class exposed (to at least one IMiD, at least one PI, and at least one anti-CD38 MoAB), at least three prior LOTs^[[1]](#footnote-2)^, ECOG score < 2, creatinine ≤ 2 mg/dL^[[2]](#footnote-3)^, and progressed within 12 months of most recent LOT. Patients must also have received at least one subsequent treatment after triple class exposure.
^2^ 47 days was the median time between apheresis and infusion in CARTITUDE-1. This criterion was applied to avoid survivorship bias in favor of cilta-cel when the treated population of CARTITUDE-1 was included in the comparative analyses.
^3^ Refractory status, cytogenetic profile, International Staging System stage, time to progression on last regimen, number of prior LOTs, years since MM diagnosis, and age.
**Abbreviations:** ATT, average treatment effect in the treated; ECOG, Eastern Cooperative Oncology Group; Hgb, hemoglobin; IMiD, immunomodulatory drug; IPTW, inverse probability of treatment weighting; LDH, lactate dehydrogenase; LOT, line of therapy; MM, multiple myeloma; MoAB, monoclonal antibody; OS, overall survival; PD, progressive disease; PFS, progression-free survival; PI, proteasome inhibitor; RW, real-world; SCT, stem cell transplant; TTNT, time to next treatment

Figure S1: Balance of covariates before and after ATT weighting for the base case


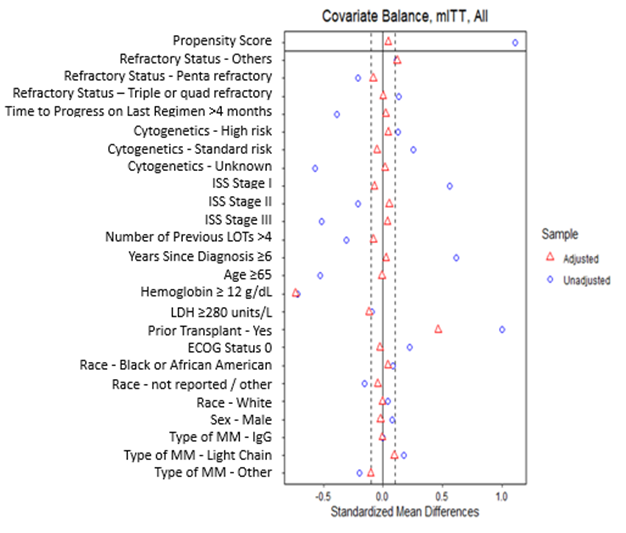


Note: the vertical dashed lines correspond to SMD=-0.1 and SMD=0.1
Penta refractory was defined as refractory to at least two IMiDs, two PIs, and an anti-CD38 MoAB; triple or quad refractory was defined as refractory to two IMiDs and one PI; or two PIs and one IMiD; or two IMiDs and two PIs. High risk cytogenetics included at least one of del17p, t(14;16), and t(4;14).
**Abbreviations:** ATT, average treatment effect in the treated; ECOG, Eastern Cooperative Oncology Group; IMiD, immunomodulatory drug; ISS, International Staging System; LDH, lactate dehydrogenase; LOTs, lines of therapy; MM, multiple myeloma; MoAB, monoclonal antibody; PI, proteasome inhibitor; SMD, standardized mean difference

**References**

1. Berdeja JG, Madduri D, Usmani SZ, Jakubowiak A, Agha M, Cohen AD, et al. Ciltacabtagene autoleucel, a BCMA-directed CAR T-cell therapy in patients with relapsed/refractory multiple myeloma (CARTITUDE-1): a phase 1b/2 open-label study (Epub ahead of print). Lancet. 2021. doi: 10.1016/S0140-6736(21)00933-8.

2. Usmani S, Ahmadi T, Ng Y, Lam A, Desai A, Potluri R, et al. Analysis of real-world data on overall survival in multiple myeloma patients with≥ 3 prior lines of therapy including a proteasome inhibitor (PI) and an immunomodulatory drug (IMiD), or double refractory to a PI and an IMiD. Oncologist. 2016;21(11):1355-61.

3. Caers J, Paiva B, Zamagni E, Leleu X, Bladé J, Kristinsson S, et al. Diagnosis, treatment, and response assessment in solitary plasmacytoma: updated recommendations from a European Expert Panel. J Hematol Oncol. 2018;11(1):10.

1. CARTITUDE-1 inclusion criteria required at least three prior LOTs *or* double refractoriness to an IMiD and a PI; however, all enrolled patients received at least three prior LOTs. [↑](#footnote-ref-2)
2. CARTITUDE-1 inclusion criterion was creatinine clearance of ≥40 mL/min/1.73 m^2^; however, all enrolled patients had creatine levels ≤ 2 mg/dL. [↑](#footnote-ref-3)
